# From inflammaging to healthy aging by dietary lifestyle choices: is epigenetics the key to personalized nutrition?

**DOI:** 10.1186/s13148-015-0068-2

**Published:** 2015-03-25

**Authors:** Katarzyna Szarc vel Szic, Ken Declerck, Melita Vidaković, Wim Vanden Berghe

**Affiliations:** Lab Protein Science, Proteomics and Epigenetic Signaling, Department of Biomedical Sciences, University Antwerp, Campus Drie Eiken, Universiteitsplein 1, 2610 Wilrijk, Belgium; Department of Molecular Biology, Institute for Biological Research, University of Belgrade, Bulevar Despota Stefana 142, 11060 Belgrade, Serbia

**Keywords:** Phytochemicals, Oxidative stress, Inflammation, Aging, Epigenetics, Metabolism, Personalized nutrition

## Abstract

The progressively older population in developed countries is reflected in an increase in the number of people suffering from age-related chronic inflammatory diseases such as metabolic syndrome, diabetes, heart and lung diseases, cancer, osteoporosis, arthritis, and dementia. The heterogeneity in biological aging, chronological age, and aging-associated disorders in humans have been ascribed to different genetic and environmental factors (i.e., diet, pollution, stress) that are closely linked to socioeconomic factors. The common denominator of these factors is the inflammatory response. Chronic low-grade systemic inflammation during physiological aging and immunosenescence are intertwined in the pathogenesis of premature aging also defined as ‘inflammaging.’ The latter has been associated with frailty, morbidity, and mortality in elderly subjects. However, it is unknown to what extent inflammaging or longevity is controlled by epigenetic events in early life. Today, human diet is believed to have a major influence on both the development and prevention of age-related diseases. Most plant-derived dietary phytochemicals and macro- and micronutrients modulate oxidative stress and inflammatory signaling and regulate metabolic pathways and bioenergetics that can be translated into stable epigenetic patterns of gene expression. Therefore, diet interventions designed for healthy aging have become a hot topic in nutritional epigenomic research. Increasing evidence has revealed that complex interactions between food components and histone modifications, DNA methylation, non-coding RNA expression, and chromatin remodeling factors influence the inflammaging phenotype and as such may protect or predispose an individual to many age-related diseases. Remarkably, humans present a broad range of responses to similar dietary challenges due to both genetic and epigenetic modulations of the expression of target proteins and key genes involved in the metabolism and distribution of the dietary constituents. Here, we will summarize the epigenetic actions of dietary components, including phytochemicals, and macro- and micronutrients as well as metabolites, that can attenuate inflammaging. We will discuss the challenges facing personalized nutrition to translate highly variable interindividual epigenetic diet responses to potential individual health benefits/risks related to aging disease.

## Review

Since people of the twenty-first century live longer, the challenge will be to make these added years as healthy and productive as possible. Societal and medical advances have extended the life of humans. Despite its significance for the well-being of individuals and the population as a whole, aging is a poorly understood process. Among the hallmarks of aging are genomic instability, telomere attrition, epigenetic alterations, loss of proteostasis, deregulated nutrient sensing, mitochondrial dysfunction, cellular senescence, stem cell exhaustion, and altered intercellular communication [1]. A large part of the aging phenotype is explained by an imbalance between inflammatory and anti-inflammatory networks [2,3]. Levels of inflammatory mediators typically increase with age even in the absence of acute infection or other physiologic stress. While levels are still in the sub-acute range, this age-related chronic inflammation underlies many aging-related conditions. According to the oxi-inflammaging theory, the aging process is a chronic smoldering oxidative and inflammatory stress that leads to the damage of cellular components, including proteins, lipids, and DNA, contributing to the age-related decline of physiological functions. This is especially evident in cells that regulate homeostasis, such as the nervous, endocrine, and immune systems. It explains their functional losses observed during aging, with a resulting increase in morbidity and mortality [4].

The progressive loss of physiological organismal and cellular integrity is the primary risk factor for major human pathologies, including metabolic syndrome, cancer, diabetes, cardiovascular disorders, and neurodegenerative diseases. Due to an imbalance between energy intake and expenditure, largely attributable to the increased availability of foods with high caloric content coupled with the adoption of a sedentary lifestyle, the continuing increase in obesity and metabolic disorders such as type 2 diabetes and accelerating aging population globally will remain the major contributors to cardiovascular mortality and aging disorders in the next 50 years. This emphasizes the importance of weight management and early intervention with regard to modifiable risk factors in overweight patients. To reduce the burden of cardiometabolic disorders and early onset of aging disorders, promoting exercise with a complementary diet, supplemented with bioactive phytochemicals, is expected to restore immune homeostasis and improve human health [5,6]. In the past couple of decades, evidence from prospective observational studies and clinical trials has converged to support the importance of individual nutrients, foods, and dietary patterns in the prevention and management of metabolic disorders [7-12]. With an emphasis on overall diet quality, several dietary patterns such as the Mediterranean diet, low glycemic index diet, moderately low carbohydrate intake, and vegetarian diets can be tailored to personal and cultural food preferences and appropriate calorie needs for weight control, diabetes prevention, and cardiometabolic management [11].

Although genome-wide association studies (GWAS) identified genetic variants that affect hundreds of genes related to energy metabolism involved in metabolic lifestyle diseases and aging, most variants identified so far confer relatively small increments in risk, leaving many questions about the remaining ‘missing’ heritability, although polygenic disease traits may account for some of these limitations [13-15]. In analogy to the reference human genome sequence which allowed GWAS studies, the NIH Roadmap Epigenomics Consortium generated today the largest collection of human epigenome sequences for epigenome-wide (EWAS) association studies [16]. From various epigenome-wide (EWAS) association studies, it has become clear that epigenetic changes in response to diet and environmental (stress) conditions complement genetic mutations and contribute to the development and progression of inflammaging diseases such as rheumatoid arthritis, metabolic disorders (obesity, type 2 diabetes), cardiovascular disease, and cancer [17-29]. For example, lifestyle factors and diet have a strong influence on the epigenetic regulation of key products of energy metabolism genes such as leptin (which is responsible for appetite control), insulin receptor (that plays a central role in glucose homeostasis), TNFα (considered as an adipokine because of its role in obesity-related inflammation and modulation of insulin response), and fatty acid synthase (catalyzing fatty acid synthesis) [30]. Accumulating evidence points to an epigenetic basis of the fetal origins of several adult metabolic disorders [31-35]. More particularly, some of the adverse epigenetic effects of lifestyle behaviors maybe rooted in perturbations *in utero* during pregnancy and during early postnatal life which shape the metabolic phenotype, perhaps across generations, which affect lifelong disease risk [32,36-38].

This review will focus on the epigenetic aspects of ‘inflammaging’ and whether there are windows of opportunity for nutri-epigenetic intervention with dietary lifestyle choices. Finally, challenges of personalized nutrition will be discussed to translate highly variable interindividual epigenetic diet responses to potential individual health benefits/risks related to diseases associated with aging.

### Epigenetics and aging

Striking links between organismal and cellular aging and epigenome alterations have recently been identified. Age-associated epigenetic changes involve alterations in DNA methylation patterns, posttranslational modification of histones, and chromatin remodeling [1,39]. In general, DNA is wrapped around nucleosomes, which are arranged as regularly spaced beads (147 bp DNA/nucleosome) along the DNA. Typically, nucleosomes consist of a histone (H) octamer of H2A/B, H3, and H4. The DNA bridging two adjacent nucleosomes is normally bound by the linker histone H1 and is termed linker DNA. While the core histones are bound relatively tightly to DNA, chromatin is largely maintained by the dynamic association with its architectural proteins (such as transcription cofactors and regulators, heterochromatin protein 1, and high mobility group (HMG) proteins). Before most activators of a gene access their DNA-binding sites, a transition from a condensed heterochromatin (‘solenoid-like fiber’) to a decondensed euchromatin (‘beads on a string’) structure appears to take place. Conversely, the acquisition of a more condensed heterochromatin structure is often associated with gene silencing [40]. The structural restriction of silenced chromatin on gene expression can be overcome by chromatin writer, reader, and eraser enzyme complexes that remodel nucleosomes along the DNA or reversibly modify histones (through posttranslational modifications, such as histone acetylation, phosphorylation, ubiquitylation, glycosylation, SUMOylation) and establish specific chromatin states involved in transcription [40-42]. Specific sets of histone modifications and/or variants are associated with genes that are actively transcribed or repressed, a phenomenon defined as the ‘histone code’ [40]. Based on coexisting histone marks and genome-wide ChIP-seq data available within the ENCODE consortium, principal component analysis has reduced the complexity of the histone code into different chromatin states that are associated with developmental and environmental cues [41-44].

DNA methylation is the best-known epigenetic mark [24,45,46]. It is catalyzed by two types of DNA methyltransferases (DNMTs): DNMT1 is a maintenance methyltransferase, whereas both DNMT3A and DNMT3B are *de novo* methyltransferases [47,48]. It is widely accepted that DNMT3A/B are mainly responsible for DNA methylation during development (differentiation) whereas DNMT1 maintains DNA methylation patterns during DNA replication (and cell division). The role of DNMT2 in DNA methylation is minor, its enzymology being largely directed to tRNA. DNA methylation is normally associated with gene inactivation, and it usually occurs in cytosine-phosphate-guanine (CpG) dinucleotides. Alternatively, DNA methylation of transcription factor binding sites which prevents the binding of repressor proteins can, paradoxically, induce gene activation. CpGs are normally methylated when scattered throughout the genome but are mostly unmethylated when clustered as CpG islands at the 5′ ends of many genes. Hypermethylation of CpG-rich promoters triggers local histone code modifications that result in a cellular camouflage mechanism which sequesters gene promoters away from transcription factors, causing stable silencing of gene expression. DNA methylation at CpG dinucleotides occurs upon transfer of S-adenosylmethionine (SAM) on cytosine by DNMTs. Recent results suggest that DNA methylation should be considered as a more dynamic and stochastic process, in which DNA methylation at each site is determined by the local activity of DNMTs, DNA demethylases, and DNA replication enzymes that are controlled by a dynamic network of chromatin marks [49] and signaling pathways [50,51]. For example, the inflammatory mediator prostaglandin E(2) (PGE(2)) has been shown to exert dynamic DNA methylation changes during cancer inflammation [52,53]. In mammalian cells, the fidelity of maintenance of methylation is 97% to 99.9% per mitosis, whereas *de novo* methylation is as high as 3% to 5% per mitosis, thus creating possibilities for dynamic epigenetic changes. Unavoidable errors may accumulate over time following long-term maintenance of epigenetic patterns or occurrence as a result of the accumulation of DNA lesions during aging in both nuclear and mitochondrial DNA caused by increased oxidative stress. Epigenetic errors could explain the stochastic differences in DNA methylation patterns reported in aging monozygotic twins [54,55]. Early studies described an age-associated global hypomethylation, concomitantly with hypermethylation of various tumor suppressor genes and Polycomb target genes [56]. Epigenetic changes accumulated throughout life may also result in the deterioration and reduced regeneration capacity of stem cells [57]. Although in most cases DNA methylation is a stable epigenetic mark, reduced levels of methylation are also observed during development. This net loss of methylation can either occur passively by replication in the absence of functional maintenance methylation pathways or, actively, by indirect removal of methylated cytosines. In mammals, a role for the 5-hydroxymethylcytosine (5-hmC) modification in DNA demethylation by ten-eleven translocation (TET) enzymes has been demonstrated as an intermediate in an active DNA demethylation pathway involving DNA repair and 5-hydroxymethylcytosine-specific DNA glycosylase activity [48,50,58].

Of particular interest, reactive oxygen species (ROS) and oxidative stress may affect DNA demethylation by DNA oxidation or TET-mediated hydroxymethylation [59,60]. For example, age-related increase in levels of 5-hmC in the brain can be prevented by caloric restriction or upregulation of specific endogenous anti-oxidants [61,62]. Furthermore, nutrients like ascorbic acid can promote DNA demethylation via increased activity of TET enzymes [63,64]. In another remarkable study, loss of TET2 and 5-hmC was found to strongly correlate with smooth muscle cell plasticity and the degree of injury in different models of vascular and atherosclerotic disease, in which ROS are critically involved [65]. Alternatively, ROS can influence the methylome by formation of oxidized DNA lesions. Replacement of guanine to 8-hydroxy-2′-deoxy-guanosine (8-OHdG), one of the major DNA oxidative damage by-products, substantially diminishes the binding of methyl-CpG binding proteins and DNMTs and results in heritable epigenetic changes [66-68]. As such, it may be expected that oxidized DNA lesions formed by the hydroxylation of pyrimidines, including 5-methylcytosine (5-mC), interfere with epigenetic signals related to 5-hydroxymethylcytosine (5-hmC) due to their structural similarities [69,70]. Finally, *in vitro* studies suggest that glutathione (GSH) depletion by redox changes leads to global DNA hypomethylation, possibly through the depletion of SAM [71,72].

**Figure 1:**
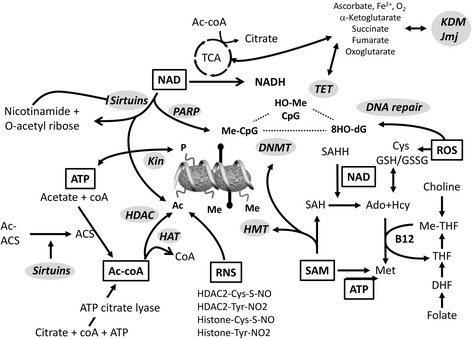
**Metabolic pathways generate essential metabolites for chromatin- and DNA-modifying enzymes.** NAD, acetyl-coenzyme A (Acetyl-coA), and S-adenosylmethionine (SAM) are elemental for epigenetic control of transcription including methylation of DNA and posttranslational modifications of histones and non-histone chromatin factors (not shown). NAD contributes to transcriptional control mainly via the activity of the protein deacetylase sirtuin, which uses NAD as one of the substrates. Sirtuins are also important for maintaining the activity of the acetyl-coA acetyltransferases. Acetyl-coA is synthesized by acetyl-coA-synthetase (ACS) and ATP-citrate lyase that use acetate and citrate as the precursors, respectively. Citrate is an intermediate/product of the TCA cycle. SAM is the methyl donor for DNA, RNA, histones, and non-histone protein methylation. S-adenosylhomocysteine (SAH) generated in each round of methylation reaction is a potent inhibitor of methyltransferases and has to be cleared by SAH hydrolase (SAHH). NAD is an essential coenzyme for SAHH. Synthesis of methionine from homocystein is achieved through extracting the methyl group from betaine, derived from choline, or 5-methyl-THF, a derivative of folic acid. Metabolism of phospholipids and folic acid may thus indirectly contribute to epigenetic regulation. Likewise, the abundance of NAD and citrate is linked to the cellular energy flux, e.g., the TCA cycle. Changes in the expression of certain genes may therefore be influenced significantly. Abbreviations used: Acetyl-coA, acetyl-coenzyme A; ACS, acetyl-coA-synthetase; AC-ACS acetylated-ACS; Ado, adenosine; HAT, histone acetyltransferase; Hcy homocysteine; MTases, methyltransferases; NAD, Nicotinamide adenine dinucleotide; ROS, reactive oxygen species, RNS, reactive nitrogen species, SAH, S-adenosyl homocysteine; TCA, tricarboxylic cycle; THF, tetrahydrofolate.

Tissues and cells of aging organisms also show age-associated changes in histone chromatin marks such as increased histone H4 lysine(K)16 acetylation, H4K20 trimethylation, or H3K4 trimethylation, as well as decreased H3K9 methylation [73-75]. Age-associated epigenomic changes could be driven by changes in expression of chromatin-modifying or -demodifying enzymes [75-77]. Of particular interest, deletion of components of histone methylation complexes (for H3K4 and for H3K27) extends longevity in nematodes and flies, respectively, and may involve the insulin/IGF-1 signaling pathway [78-81]. It is not yet clear whether aging is a cause or consequence following purely epigenetic changes or alterations affecting metabolic or signaling pathways outside of the nucleus. Importantly, since the activities of histone-modifying enzymes also depend on intracellular levels of essential metabolites (acetyl-coA, Fe, ketoglutarate, NAD^+^, S-adenosylmethionine), epigenetic changes are tightly linked to global cellular metabolism and energy levels [82-88] (Figure 1). Finally, ROS (such as ^•^O_2_, ^•^OH, H_2_O_2_, NO, and ^1^O_2_) as well as reactive nitrogen intermediates such and NO and reactive nitrogen species (RNS), produced by neutrophils, macrophages, endothelial, and other cells, can indirectly modulate the activity of the epigenetic machinery. For example, ROS were demonstrated to modulate the activity of the Rph1 demethylase specifically at subtelomeres to remodel chromatin and extend lifespan [89].

Although epigenetic modifications previously were thought to be fixed during development and maintained over the lifetime, more recent research provides evidence that epigenetic mechanisms allow rapid adaptations to a changing environment and are responsive to signaling cascades [50,51]. Therefore, epigenetic mechanisms may exacerbate the epidemic of metabolic disease by first contributing to the development of obesity and type 2 diabetes and then passing modifications on to the subsequent generation via transgenerational inheritance [90]. Nevertheless, epigenetic mechanisms might also prevent the development of type 2 diabetes through nutritional intervention therapies [12,34,91,92]. Recent success of therapeutic intervention in chronic inflammatory diseases using epigenetic modifiers such as histone deacetylase (HDAC) and DNMT inhibitors has fuelled interest in methylome profiling of complex diseases [92-103].

### Crosstalk of inflammation and energy metabolism fuel epigenetic plasticity

**Figure 2:**
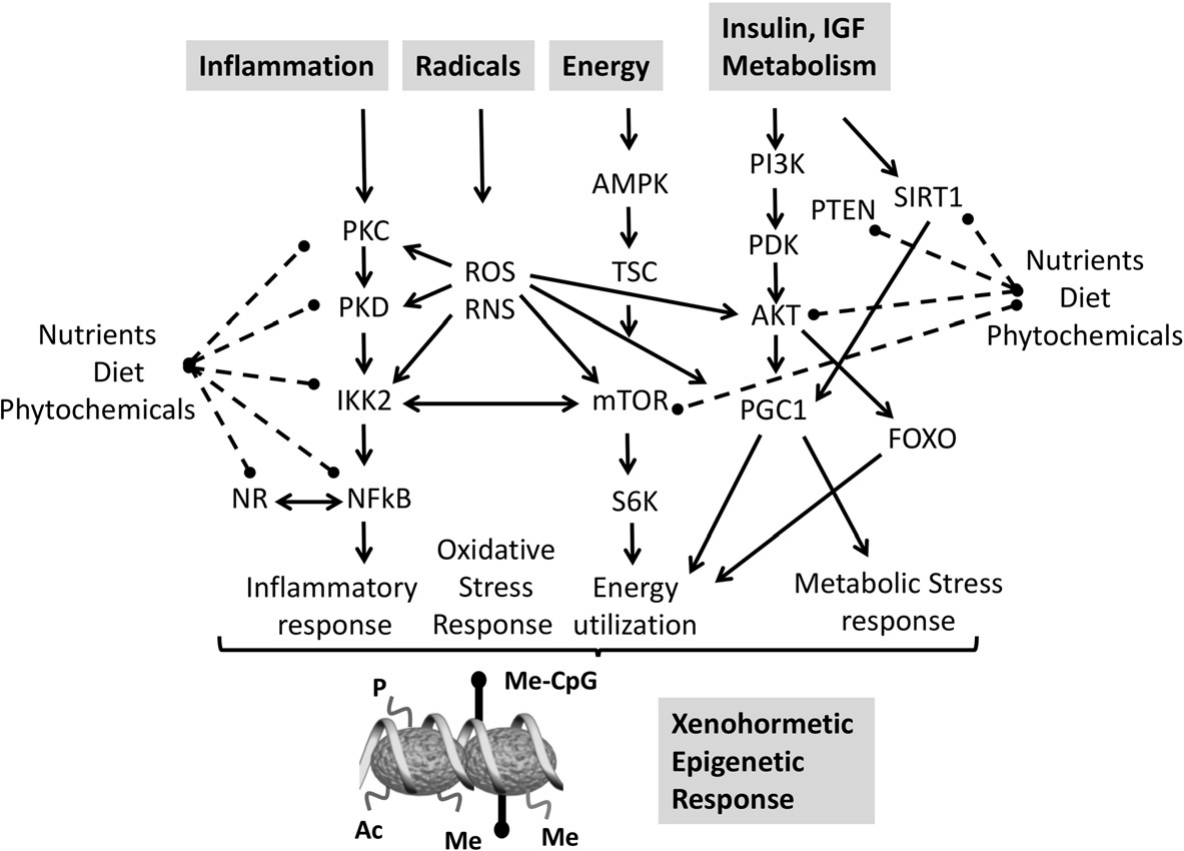
**Plant phytochemicals achieve hormesis through multifocal pathway inhibition.** Our health strongly benefits from interactions of a large number of plant molecules in our diet with key regulators of mammalian physiology (adapted from [288]). Various plant-derived molecules are synthesized as secondary metabolites in response to stress. During adversity in the context of particular environmental stresses, animals have retained the ability to sense these stress signaling molecules synthesized by their distant ancestors, through enzymes and receptors which regulate inflammation-energy-metabolism pathways to protect and to increase the survival of the organism. Abbreviations used: PKC, protein kinase C; PKD, protein kinase D, IKK2, inhibitor of IkB kinase 2; ROS/RNS, reactive oxygen/nitrogen species; NR, nuclear receptor; AMPK, AMP-activated protein kinase; TSC, tuberous sclerosis complex mTOR, mammalian target of rapamycin; R6SK ribosomal S6 kinase; PI3K, phosphoinositide 3-kinase; PDK, pyruvate dehydrogenase kinase; AKT/PKB, protein kinase B; PGC1, peroxisome proliferator-activated receptor c coactivator 1; SIRT, sirtuin; FOXO, forkhead box O.

**Figure 3:**
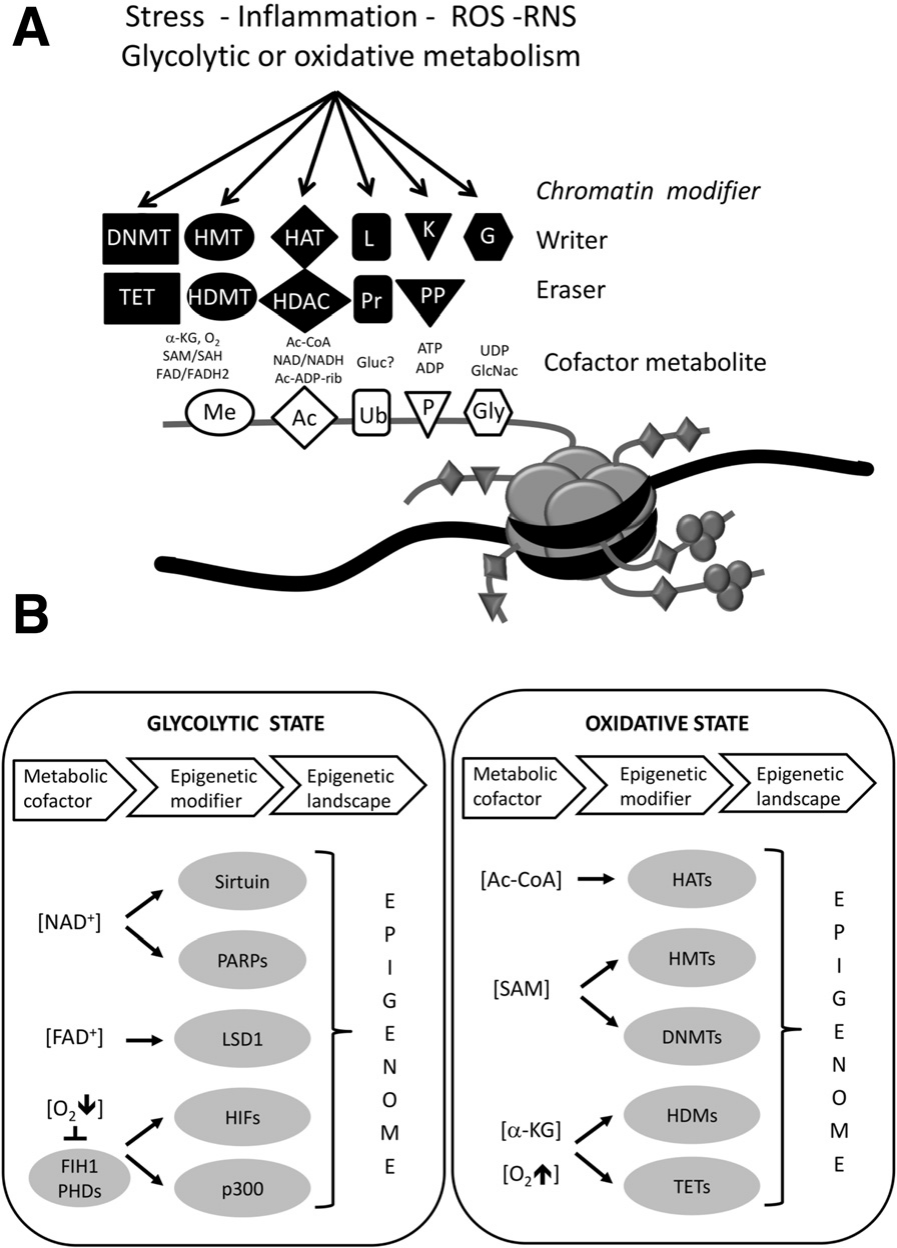
**Activity of chromatin modifying writer-eraser enzymes depends on available concentrations of cofactor metabolites and environmental signals. (A)** Schematic representation of a nucleosome with extruding histone tails with residues that can be modified by various chromatin writer (i.e., DNA methyltransferase (DNMT), histone methyltransferase (HMT), histone acetylase (HAT), ubiquitin ligase (L), kinase (K), glycosylase (G)) or chromatin eraser enzymes (i.e., DNA hydroxymethylase (TET), demethylase (HDMT), deacetylase (HDAC), proteasome (Pr), phosphatase (PP)), resulting in dynamic histone methylation (Me), acetylation (Ac), ubiquitination (Ub), phosphorylation (P), and glycosylation (Gly). These histone modifications have been associated with changes in chromatin organization, gene activation, silencing, and several other nuclear functions (adapted from [338]). **(B)** Hypothetical model of a glycolytic-oxidative metabolic switch and its possible influence on epigenetic modifiers and the epigenetic landscape (adapted from [339]).

An increasing number of experimental and epidemiological evidence links multifaceted process of aging to systemic low-grade inflammation and disturbances in cellular metabolism and protein homeostasis [104-106]. An efficient autophagic flux, i.e., cellular mechanism for the degradation and recycling of cellular components, is essential for healthy aging and maintenance of cellular homeostasis and links inflammation to metabolic disorders (Figure 2). Autophagy negatively regulates inflammasome activation by maintaining mitochondrial homeostasis. Reciprocally, mitochondrial energy metabolites also regulate aging and autophagy through as-yet-elusive metabolic circuits [105]. Inflammation also profoundly affects the metabolic bioenergetic profile of target cells, promoting aerobic glycolysis, a process called the ‘Warburg effect’, first described in tumor cells [107]. Different cell conditions require flexible metabolic programs to support unique bioenergetic demands. Metabolic pathways rely on the dynamic balance between anabolic processes to support the synthesis of cellular building blocks and catabolic processes to ensure adequate bioenergetic resources. Beyond nutrient-sensing pathways which control gene transcription and intercellular/extracellular energetic status, nutrient-responsive metabolites, such as ATP, acetyl-CoA, UDP-N-acetylglucosamine (UDP-GlcNAc), and S-adenosyl methionine, mediate crosstalk between metabolism, cellular signaling, and the epigenetic control of transcription programs [108-116] (Figure 3). By operating as indicators of metabolic status, these metabolites serve as substrates for posttranslational modifications, including acetylation, glycosylation, methylation, and phosphorylation, which regulate the activity of metabolic enzymes, signaling pathways, and transcription factors. Because histone-modifying enzymes including kinases, acetyltransferases, and methyltransferases consume key metabolites, the metabolic state of a given cell will also be reflected in the chromatin modification patterns. In this respect, changes in nuclear acetyl-CoA or NAD^+^ levels affect histone acetylation patterns [88,114]. However, the specificity of chromatin changes also depends on the gene-specific recruitment of histone-modifying enzymes to specific chromosomal domains via their interaction with DNA-binding factors, ncRNAs [117-119]. Also, enzymes that use the same metabolite but modify different substrates, such as DNA or histone methyltransferases, may compete with each other leading to either one or the other methylation product. Furthermore, many nutrient metabolites have been shown to have a direct effect on gene expression patterns through binding to nuclear receptors that in turn affect the transcription of the gene they bind to [120]. Interestingly, even transient changes in the nutrition can have a long-lasting impact on gene expression patterns. Heritable ‘memory’ effects of metabolic disturbances have been demonstrated by the ablation of key epigenetic enzymes such as SIRT1, HDAC6, and KDM3A in models of metabolic disorders [114,116]. These findings pave the way to the development of therapeutic strategies against epigenetic modifier enzymes for the treatment of metabolic and aging disorders [121-123]. Recent theories propose that mitochondria and energy metabolism play a major role in the regulation of health span through Krebs cycle intermediates that shape the epigenetic landscape of chromatin by regulating DNA and histone methylation during the aging process [124,125] (Figure 3B). Of particular interest, the histone variant MacroH2A1.1 but not MacroH2A1.2 was found to bind with high affinity to the SIRT1-metabolite O-acetyl ADP ribose. Upon its overexpression, it ameliorates glucose metabolism and reduces expression of lipidogenic genes and fatty acids [126]. In another study, genetic ablation of histone macro-H2A1 resulted in increased leanness, glucose tolerance, and energy expenditure in mice fed with a high-fat diet [127]. Major metabolic changes are also observed in cancers [72,88,128,129]. The ‘Warburg effect’ is accompanied by major alterations in gene expression profile whose causes are likely to be associated with specific chromatin-remodeling events [130-133]. Furthermore, mutated isoforms of the core metabolic enzymes isocitrate dehydrogenase (IDH), succinate dehydrogenase (SDH), and fumarate hydratase (FH) result in accumulation of particular metabolites which inhibit TET enzymes responsible for oxidizing 5-mC, leading to pervasive DNA hypermethylation [111,134-136]. In analogy to ‘oncometabolites’ whose accumulation triggers aberrant signaling resulting in initiation of carcinogenesis, depletion of ‘gerometabolites’ was found to drive aging [137,138]. Altogether, the cellular metabolism is tightly regulated, and imbalance of energy intake and expenditure contribute to metabolic diseases, cardiovascular diseases, cancer, and other aging diseases. Dynamics and/or reversibility of epigenomic changes in response to altered metabolic states needs to be further investigated.

### Nutri-epigenomics: lifelong remodeling of our epigenomes by nutritional, phytochemical, and metabolic factors

**Figure 4:**
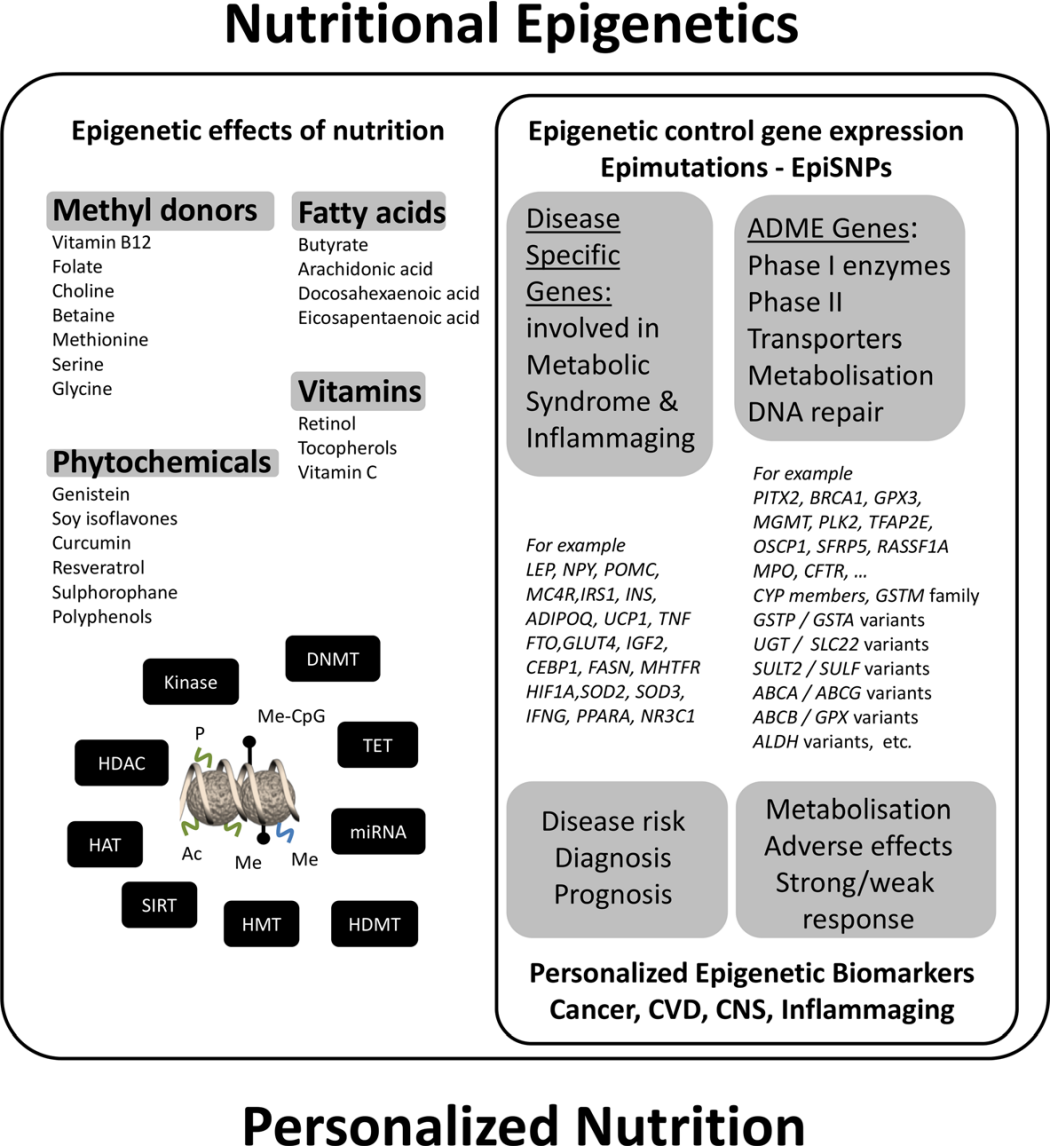
**Overview of the mechanisms and consequences of epigenetic regulation by nutritional compounds.** Modulation of different classes of chromatin writers-erasers by phytochemicals (left panel). Genes encoding absorption, distribution, metabolism, and excretion (ADME) proteins can be epigenetically regulated and thereby determine individual nutritional responses. Epigenetic modification of disease-related genes can contribute to diagnosis (biomarker) as well as disease prevention or progression (right panel).

Phytochemicals from plants appear to be crucial to achieve the correct relationship between man and nature - between dietary balance and health (Figure 4). Several polyphenolic compounds, such as resveratrol, tea catechins, and flavonoids, which are commonly found in vegetables, fruits, and plant-derived juices or beverages, exert well-evidenced cardioprotective, neuroprotective, chemopreventive, and anti-inflammatory properties, but, nevertheless, further clinical and epidemiological research is required. Classic proposed mechanisms for the health benefits of phytochemicals are the following: (1) direct antioxidant activity or increase in the expression of antioxidant proteins; (2) attenuation of endoplasmic reticulum stress signaling; (3) blockade of pro-inflammatory cytokines; (4) blockade of transcription factors related to metabolic diseases; (5) induction of metabolic genes expression; and (6) activation of transcription factors that antagonize inflammation [139]. Rather than the chemical conversion of food to energy and body matter of classic metabolism, food is now also a conditioning environment that shapes the activity of the (epi)genome and determines stress adaptive responses, energy metabolism, immune homeostasis, and the physiology of the body [91,140-143]. Human epidemiological studies and appropriately designed dietary interventions in animal models have provided considerable evidence to suggest that maternal nutritional imbalance and metabolic disturbances, during critical time windows of development, may have a persistent effect on the health of offspring and may even be transmitted to the next generation [22,144-149]. This has led to the hypothesis of ‘fetal programming’ and new term ‘developmental origin of health and disease’ (DOHaD) [35,150]. This hypothesis postulates that a nutritional or environmental mismatch between prenatal (*in utero* gestation) and postnatal life (weaning, infancy, adult life), plays an important causative role in non-communicable diseases, including diabetes, cardiovascular disease, allergy, some forms of cancer, cognitive decline, and affective disorders [21,146,151-156]. The various non-Mendelian features of metabolic disease, cancer, or chronic inflammatory disorders, clinical differences between men and women or monozygotic twins, and fluctuations in the course of the disease are consistent with epigenetic mechanisms in the influence of fetal and/or lifelong nutrition or stochastic events on adult phenotype [22,144-149,157-159].

Thus, lifetime shapes the multitude of epigenomes not only within but also across generations [22,35,148,160-162]. Interest in transgenerational epigenetic effects of food components has initially been fuelled by observations in Agouti (A^vy^/a) mice fed with a soy polyphenol genistein (GEN), which revealed changes in coat color, related to epigenetic changes in DNA methylation patterns in their offspring and protection against diabetes, obesity, and cancer across multiple generations [163-165]. However, some of these findings were contested in more recent studies with A^vy^/a mice fed with soy protein isolate, containing comparable amounts of genistein [166]. In another study by Rosenfeld and colleagues, no association between a genistein-based diet and the so-called pseudoagouti, brown phenotype was revealed [167]. Also, only weak transgenerational effects could be observed with soy polyphenols in *Daphnia Magna*, despite the presence of functional DNMTs [168]. Nevertheless, the honeybee (*Apis mellifera*) is probably the clearest example of induction of alternative phenotypes and aging epigenotypes by nutrition in early life [169]. Female bees are genetic clones. However, queens are distinct from workers in their morphology, capacity to reproduce, behavior, and longevity. The difference between the queen and worker castes lies in the exposure of the genetically identical larvae to royal jelly, an as yet incompletely defined mixture of proteins, amino acids, vitamins, fatty acids, steroids, hormones, lipids, and other nutrients [170-176].

Studies of human populations following famine have suggested that pathologies in later life are dependent on the timing of nutritional insult during pregnancy. Follow up of the Dutch Hunger Winter cohort showed that cardiovascular disease was more prevalent in offspring of mothers who were severely undernourished during the first trimester of their pregnancies in 1944 to 1945, as compared to those born to mothers whose pregnancies were more advanced at the time of nutritional insult [177-179]. Also, paternal patterns of nutrition during the prepubertal growth period in children in Överkalix, in Sweden, during the nineteenth century are associated with differential risk of early cardiovascular death in their grandchildren [180,181]. Today, various epigenetic changes have already been characterized which are involved in atherogenesis [21,22,182-185]. Hypercholesterolemia, obesity, hyperhomocysteinemia, and high glucose are important cardiovascular disease risk factors which are implicated in enhanced inflammatory signaling, and long-lasting effects are driven by epigenetic reprogramming, which promote differentiation of monocytes/macrophages into more proatherogenic phenotypes [186-192]. Recent evidence suggests that the pathogenetic role of hyperhomocysteinemia in vascular diseases might be mediated via adenosyl-homocystein (Hcy) accumulation and DNA methylation. Hcy competes with SAM (the methyl-group donor) for binding on DNMT, which may lead to passive loss of methylation in replicating DNA. High blood Hcy levels correlate with DNA hypomethylation and atherosclerosis and can lead to a 35% reduction in DNA methylation status of peripheral blood lymphocytes [193-196]. Similarly, insulin, glucose, folate, or flavanol-rich diets interfere with the methyl donor metabolism and the available pool of SAM, resulting in DNA methylation changes [196-199]. In contrast, very few studies have focused on impact of dietary methyl donors on histone methylation, which is also affected by alterations in SAM/S-adenosylhomocysteine (SAH) ratios [193,200]. As such, specific dietary classes of functional food maybe designed as therapeutic epigenetic modulators in lifestyle disease, such as metabolic disorders (diabetes), cardiovascular disease, asthma/COPD, and rheumatoid arthritis [91,142,143,201,202].

Epidemiologic and medical anthropological studies have indicated that flavanol-rich diets are inversely associated with cardiovascular risk [203-209]. Locus-specific DNA methylation changes, both hyper- and hypomethylation, have been demonstrated at the promoter of several genes involved in the pathogenesis of atherosclerosis, such as extracellular superoxide dismutase (SOD), hormone receptors (glucocorticoid receptor (GR), estrogen receptor (ER), peroxisome proliferator-activated receptor (PPAR), arylhydrocarbon receptor (AhR), liver X receptor (LXR)), endothelial and inducible nitric oxide synthase (iNOS/eNOS), 15-lipoxygenase (LOX), fibroblast growth factor (FGF)2, hypoxia-inducible factor (HIF)1α, myc, insulator CCCTC binding factor (CTCF), and metalloproteases (MMPs) [189,210-213]. In a proatherogenic murine model, DNA-methylation polymorphisms preceded the appearance of histological signs of atherosclerosis [187,188]. Interestingly, involvement of the inducible JMJD3 demethylase was demonstrated to regulate monocyte/macrophage transdifferentiation programs, illustrating that developmental programs are plastic and monocyte lineage differentiation is susceptible to inflammatory pathways and oxidative stress [214]. A role for the JMJD1A demethylase was demonstrated in metabolic gene expression and obesity resistance [215]. Furthermore, it was found that knockdown of the LSD1 demethylase affected monocyte adherence in a proatherogenic diabetic mouse model [216]. This suggests that LSD1 contributes to metabolic memory through long-term changes in gene expression via alterations in chromatin structure [217,218].

Poor maternal nutrition has also been associated with increased risk of type 2 diabetes over several generations in North American Indians [219,220]. Individuals with metabolic syndrome, obesity, type 2 diabetes, and cardiovascular disease may show a lifelong imbalance between energy intake and expenditure due to incorrect epigenetic programming during their early development as a result of placental insufficiency, inadequate maternal nutrition, metabolic disturbances, or neonatal medication [145,219-224].

Recently, evidence emerged that also timing (preconception, pregnancy, lactation, neonatal life, early life, pre-/post-menopause, puberty) of various dietary exposures may be vitally important in determining health beneficial effects, as epigenetic plasticity changes continually from conception to death [225]. In principle, epigenetic changes occurring during embryonic development will have a much greater impact on the overall epigenetic status of the organism because, as they can be transmitted over consecutive mitotic divisions, alterations occurring in single embryonic stem cells will affect many more cells than those occurring in adult stem and/or somatic cells during postnatal development [147]. Epigenetic plasticity further also depends on other processes such as chromosomal instability, telomere shortening, metabolic cycles, mitochondrial deteriorations, and oscillatory, circadian, or seasonal rhythms of systemic hormone levels (hypothalamic-pituitary-adrenal (HPA) axis) [21,22,93,224-228]. In addition to epigenetic imprinting during crucial periods of development, stochastic or genetically and environmentally triggered epigenomic changes (epimutations) occur day after day and accumulate over time, as maximal differences in DNA methylation profiles are observed in aged monozygotic twins with a history of non-shared environments [55,96]. Concerning nutritional transgenerational inheritance, there is increasing evidence in both plants and animals that, following nutritional intervention (caloric, iron and protein restriction, polyphenol-, folate-, micronutrient-, fat-, or carbohydrate-rich diet), maternal diabetes, during pregnancy, and lactation, can affect the following generation(s) [148,153,164,165,229-231]. Although it has long been thought that the epigenomic profile is wiped clean in the embryo shortly after fertilization, with the exception of imprinted genes, methylation clearing is not complete after fertilization and on a global DNA level is reduced to 10% [232,233] or converted into hydroxymethylcytosine [234]. Alternatively, it cannot be excluded that transgenerationally inherited nutritional effects may also depend on Polycomb proteins [148,235-237], miRNAs, or long noncoding RNAs [19,238-242]. Since hsp90 inhibitors trigger previously hidden morphological phenotypes in the next generation and for several generations thereafter, increasing evidence also supports a ‘capacitor’ role (i.e., storage of accumulated stress) of hsp90 in buffering transgenerational epigenetic variation during environmental or nutritional stress [243-245].

A next challenge will be to determine which adverse epigenomic marks are reversible by specific diets, drugs, or lifestyle changes [22,116,142,143,146,201,225,231]. Numerous botanical species and plant parts contain a diverse array of polyphenolic phytochemicals which exert health-beneficial effects in man by their anti-inflammatory, anti-oxidant, phytohormone, cardio-protective, cancer preventive, and anti-bacterial properties, by maintaining immune homeostasis (hormesis) [246,247]. Phytochemicals have also successfully been applied for regenerative medicine and cancer stem cell therapy [248-253]. Oxidative stress and inflammatory damage play an important role in epigenetic reprogramming of expression of cytokines, oncogenes, and tumor suppressor genes, thereby setting up a ground for chronic inflammatory diseases and carcinogenesis [254-256]. As such chemoprevention, the strategy to inhibit, retard, or even reverse the epigenetic stage of chronic inflammation is one of the most rational approaches to reduce the global burden of non-communicable aging diseases [30,153,256,257].

Today, various nutritional compounds (including epigallocatechin gallate, resveratrol, genistein, curcumin, isothiocyanates, withaferin A) have been characterized which interfere with enzymatic activity of chromatin writers, readers, or erasers such as DNMT, class I to IV histone deacetylases (HDACs), histone acetyl transferases (HATs), and class III HDAC sirtuins (SIRTs) which modulate inflammatory responses and immunological senescence ([91,140,141,146,155,231,258-269] and references included) (Figure 4). HDACs are zinc metalloproteins which rely on Zn^2+^ for their activity and are divided into four classes based on their homology with yeast HDACs. Class III HDACs, called sirtuins, are zinc independent but nicotinamide adenine dinucleotide (NAD^+^) dependent. Class I to IV HDAC inhibitors characteristically contain a Zn^2+^ chelating group consisting of a thiolate, thiol, hydroxamate, carboxylate, mercaptoamide, epoxide, or ketone group. Natural HDAC inhibitors can be divided in following groups based on their chemical characteristics: carboxylates, organosulfides, isothiocyanates, hydroamates, cyclic tetrapeptides, and macrocyclic depsipeptides [261]. In contrast to natural HDAC inhibitors, only few natural products (i.e., niacin, dihydrocoumarin) have been identified as inhibitors of class III HDACs. Reciprocally, various natural flavonoids have been identified as activators of class III HDACs (SIRTs). Finally, turmeric and green tea have been identified as sources of natural inhibitors of p300/CBP HAT. Finally, DNMT inhibitors work mainly through one of the following mechanisms, either covalent trapping of DNMT through incorporation into DNA (i.e., nucleoside analogs decitabine, 5-azacytidine), non-covalent blocking of DNMT catalytic active site (i.e., EGCG, parthenolide), interruption of binding site of DNMT to DNA (i.e., procaine), degradation of DNMT (i.e., decitabine), or suppression of DNMT expression (i.e., miRNAs). Furthermore, a number of natural compounds act as multifunctional ligands by simultaneously acting on nuclear hormone receptors and changing activity of histone-modifying enzymes and DNMTs [270-274]. Although health-protective anti-oxidant or anti-inflammatory effects of dietary factors and extracts have frequently been demonstrated in *in vitro* experiments at concentrations which can never be achieved *in vivo*, ‘epigenetics’ might shed a more realistic light on dietary studies, as long life exposure at physiological concentrations could lead to remodeling of the epigenome in a cumulative fashion by repetitive effects on the epigenetic machinery [160,161,275]. Particular attention needs to be given to natural compounds which can trigger opposite effects on HDAC/HAT/DNMT or histone (de)methylase (H(D)MT) depending on the concentration- or cell type-specific metabolization [260,261]. It should also be stressed that it is not known whether all of them can be considered authentic epigenetic modifiers because it has not yet been demonstrated whether the epigenetic modifications which they induce are stable over time. Interestingly, even transient exposure to a specific dietary component can induce long-lasting epigenetic changes in inflammatory gene expression [218,276]. Alternatively, compounds may chemically interfere with histone mark interacting protein structure motifs (such as chromo-, bromo-, or tudor domains) [277-279].

Besides specific interference of the diet with chromatin-modifying enzymes and DNMTs at particular target genes, global epigenetic changes can also occur following biochemical metabolization of dietary factors, which can deplete cellular pools of acetyl-CoA, NAD^+^, and methyl donors, resulting in unbalanced DNA methylation and/or protein acetylation or methylation [87,266,280]. For example, diets lacking in substrate or cofactors in methyl donor metabolism can contribute to DNA hypomethylation by impairing synthesis of SAM [194]. This methylation cycle is frequently cited to explain relations between diet and epigenetic changes [193,281]. However, even without nutritional deficiency of methyl groups, impaired synthesis of SAM and perturbed DNA methylation can happen when the need for glutathione (GSH) synthesis increases [282]. Diets or nutritional compounds which affect energy metabolism or mitochondrial respiration can have global epigenetic effects upon changes in NAD^+^ availability and SIRT activity [283]. Since SIRT activation has been linked to longevity (increased lifespan and healthy aging) and mimics a caloric restricted diet, SIRT activators such as resveratrol represent a major class of caloric mimetic phytochemicals which could reverse metabolic disease [280,284-286].

### Xenohormetic epigenetic effects of plant secondary metabolites across species: evolutionary role for stress adaptive responses in healthy aging and longevity

The xenohormesis hypothesis proposes that under stressful conditions, plants synthesize phytochemicals (xenohormetins), which, when incorporated into the heterotroph diet, induce defense responses, leading to an extended lifespan [287]. Most plants contain toxic molecules, in order to prevent pathogen colonization and insect-mediated damage and also to discourage animals from eating them. According to an evolutionary theory of stress adaptation, animals and fungi (heterotrophs) have evolved the ability to respond to stress-induced chemical molecules related to the status of its environment or food supply from other species, to prime a defense response that increases its chances of survival upon subsequent environmental stress challenges. Xenohormesis suggests that the majority of health benefits from phytochemical consumption do not result from responses to mild cellular damage or from their antioxidant properties but rather from the evolutionarily adaptive modulation of the enzymes and receptors of stress-response pathways in mammals [288]. Therefore, these phytochemicals, working as interspecies transference signals, are preparing living beings for adversity [287]. According to this model, animals facing reduced food availability or other biological stresses have a selective advantage to divert limited resources away from reproduction and growth into maintenance and defense until their offspring have a better chance of survival. Similarly, lifespan extension through caloric restriction may have evolved to promote survival in an environment with poor prospects for reproduction. Also, fasting on alternate days shares similar health benefits as caloric restriction. Perhaps it mimics a natural circumstance in which increasing food uncertainty prepares for possible future starvation conditions. For example, various environmental stress-induced secondary plant metabolites such as resveratrol, butein, and fisetin can induce defense responses in fungi, nematodes, flies, fish, and mice or can extend lifespan by mimicking ‘caloric restriction’ [288]. These chemical signals regulate the epigenome by modulating metabolic pathways and function of chromatin-modifying enzymes as well as transcription factors that are responsible for recruiting these enzymes.

### Interindividual epigenetic variation in diet responses and challenges of personalized nutrition

From clinical and diet intervention studies, it appears that individuals display different responses to pharmacological nutritional interventions, respectively, that result in variable benefits to particular treatments [143,289,290]. Similarly, considerable heterogeneity can be observed in biological aging and chronological age is not a reliable marker for healthy aging [291]. Heterogeneity in responsiveness can obscure associations between dietary intakes and health outcomes and bias the identification of the effects of bioactive phytochemicals in specific subpopulations.

Pharmacogenomic and -kinomic studies demonstrate that for some drugs and/or bioactive nutrients, individuals can be categorized into poor, intermediate, or extensive absorbers or metabolizers and dosing has to be personalized [102,143,160,161,203,292-295]. Various genetic single-nucleotide polymorphisms (SNPs) with known relevance to drug pharmacokinetics, such as detoxification enzymes and transporters, have already been compiled in online databases. For example, several genetic variants exist for genes encoding glutathione S-transferases (GSTs), which play major roles in the metabolism of glucosinolates and bioavailability of isothiocyanates that are present in cruciferous vegetables (broccoli) [296,297]. A significant interindividual variation has also been described for the LDL-cholesterol lowering response to plant sterol consumption, and it is associated with *ABCG8* gene polymorphism [298].

However, this is still insufficient to explain the large interindividual variations in therapeutic responses. In recent years, evidence that has accumulated suggests that epigenetic aberrations of key ADME genes (genes related to drug absorption, distribution, metabolism, and excretion) involved in the metabolism and distribution of phytochemicals also contribute to interindividual variations in the nutritional response [102,299]. For example, hypermethylation of ADME gene promoters has been observed in cancer tissue, resulting in gene repression of various phase I and II enzymes, including CYP450s and UDP-glucuronosyltransferases, as well as ABC efflux transporters [300-302] (Figure 4). The introduction or removal of CpG dinucleotides at SNPs (CpG-SNPs, epimutations) may represent a potential mechanism through which SNPs affect gene function via epigenetic processes [31,303]. Conversely, epigenetic changes could increase susceptibility to genetic point mutations [304]. This indicates a complex interrelationship between genetic and epigenetic variations in different diet-related disease phenotypes [31,304-309]. Personalized nutrition is an increasingly recognized paradigm in nutrition research. Therefore, some population subgroups may gain more benefit than others from the consumption of plant foods and their bioactives. The further determination of environmental factors responsible for interindividual variations in the endocrine system, epigenetic profiles, and microbiome communities and the identification of ‘susceptibility profiles’ in response to plant bioactive consumption could lead to targeted dietary advice and use of functional foods customized for different population subgroups [143,310-312]. In contrast to prominent quantitative epigenetic changes at tumor suppressor genes (>60% increase of DNA methylation) associated with cancer, more subtle epigenetic changes are typically observed in cardiometabolic disorders (<20%) [312-320]. To reverse such subtle changes, several nutrients and bioactive food compounds may be preferred over toxic antineoplastic epigenetic drugs [91,121,142,143,321-327]. This will encourage the characterization of robust epigenetic dietary biomarkers and design of functional foods that could help to combat or prevent inflammaging-related metabolic diseases.

## Conclusions

The phenotype of an individual is the result of complex ongoing gene-environment interactions in the present, past, and ancestral environments, responsible for lifelong remodeling of our epigenomes. In recent years, several studies have demonstrated that disruption of epigenetic mechanisms can alter immune function and that epimutations not only contribute to certain cancers but also to lifestyle diseases such as type 2 diabetes, allergies, cardiovascular disease, and rheumatoid arthritis, as well as unhealthy aging. Various replication-dependent and -independent epigenetic mechanisms are involved in developmental programming, a lifelong intertwined process of monitoring and responding to environmental changes, and the transmission of transgenerational effects. It is likely that improved understanding of epigenetic processes will allow us to manipulate the epigenome which represents a reversible source of biological variation [328,329]. We believe that herein resides a great potential for chemoprevention, alleviation of chronic inflammatory disorders, and healthy aging. Much attention is currently focused on the modulation of hyper/hypomethylation of key inflammatory genes by dietary factors as an effective approach to chronic inflammatory disease management and general health benefits [146,155,231,259-266]. In this respect, ‘Let food be your epigenetic medicine’ could represent a novel interpretation of what Hippocrates said twenty-five centuries ago. As such, it will be a challenge for future nutritional research to identify novel epigenetic targets that promote healthy aging [247,330-335]. Given several encouraging trials, prevention and therapy of age- and lifestyle-related diseases by individualized tailoring of optimal epigenetic diets or supplements are conceivable. However, these interventions will require intense efforts to identify health beneficial relationships in intra- (age/tissue/cell-type specific) and interindividual variation of epigenetic, genetic, and environment interactions [35,310,336,337].

In conclusion, ‘inflammaging’ disorders as well as dietary lifestyle reveal a dazzling complexity of epigenetic changes during lifetime. To prevent or to reverse adverse epigenetic alterations associated with multifactorial aging diseases, combinatorial therapeutic and/or nutritional approaches will be necessary to modulate different classes of chromatin modifiers. Future research needs to evaluate the optimal dose and exposure window during gestation *in utero*, post-natal early life, prepuberty, and adult life for specific dietary composition to elicit maximal epigenetic benefits against inflammaging and improve the overall quality of life of the human population [35,309,324-327].

## Abbreviations

5-hmc: 5-hydroxymethylcytosine
5-mC: 5-methylcytosine
8-OHdG: 8-hydroxy-2′-deoxy-guanosine
ADME: absorption, distribution, metabolism, excretion
AhR: arylhydrocarbon receptor
CpG: cytosine-phosphate-guanine
CTCF: insulator CCCTC binding factor
DNMT: DNA methyltransferase
DOHD: developmental origin of health and disease
eNOS/iNOS: endothelial and inducible nitric oxide synthase
ER: estrogen receptor
FGF: fibroblast growth factor
FH: fumarate hydratase
GR: glucocorticoid receptor
GSH: glutathione
HAT: histon acetyl transferases
HDAC: histone deacetylase
HIF: hypoxia-inducible factor
HMT: histone methyltransferases
HPA: hypothalamic-pituitary-adrenal
IDH: isocitrate dehydrogenase
IGF: insulin growth factor
JMJD: jumonji domain
KDM: lysine demethylase
LOX: lipoxygenase
LXR: liver X receptor
MMP: metalloproteases: ncRNAs: noncoding RNA
PDK: pyruvate dehydrogenase kinase
PGE2: prostaglandin E2
PPAR: peroxisome proliferator-activated receptor
RNS: reactive nitrogen species
ROS: reactive oxygen species
SAM: S-adenosylmethionine
SDH: succinate dehydrogenase
SIRT: sirtuin
SNP: single nucleotide polymorphism
SOD: superoxide dismutase
TET: ten-eleven translocation
UDP-GlcNAc: UDP-N-acetylglucosamine
